# Methyl-Hydroxylamine as an Efficacious Antibacterial Agent That Targets the Ribonucleotide Reductase Enzyme

**DOI:** 10.1371/journal.pone.0122049

**Published:** 2015-03-17

**Authors:** Esther Julián, Aida Baelo, Joan Gavaldà, Eduard Torrents

**Affiliations:** 1 Departament de Genètica i de Microbiologia, Facultat de Biociències, Universitat Autònoma de Barcelona, Bellaterra, Spain; 2 Institute for Bioengineering of Catalonia (IBEC), Bacterial infections and antimicrobial therapies; Baldiri Reixac 15-21, Barcelona, Spain; 3 Infectious Diseases Research Laboratory, Infectious Diseases Department, Vall d’Hebron Research Institute, Hospital Universitari Vall d'Hebron, Barcelona, Spain; Queen's University Belfast, UNITED KINGDOM

## Abstract

The emergence of multidrug-resistant bacteria has encouraged vigorous efforts to develop antimicrobial agents with new mechanisms of action. Ribonucleotide reductase (RNR) is a key enzyme in DNA replication that acts by converting ribonucleotides into the corresponding deoxyribonucleotides, which are the building blocks of DNA replication and repair. RNR has been extensively studied as an ideal target for DNA inhibition, and several drugs that are already available on the market are used for anticancer and antiviral activity. However, the high toxicity of these current drugs to eukaryotic cells does not permit their use as antibacterial agents. Here, we present a radical scavenger compound that inhibited bacterial RNR, and the compound's activity as an antibacterial agent together with its toxicity in eukaryotic cells were evaluated. First, the efficacy of N-methyl-hydroxylamine (M-HA) in inhibiting the growth of different Gram-positive and Gram-negative bacteria was demonstrated, and no effect on eukaryotic cells was observed. M-HA showed remarkable efficacy against *Mycobacterium bovis* BCG and *Pseudomonas aeruginosa*. Thus, given the M-HA activity against these two bacteria, our results showed that M-HA has intracellular antimycobacterial activity against BCG-infected macrophages, and it is efficacious in partially disassembling and inhibiting the further formation of *P*. *aeruginosa* biofilms. Furthermore, M-HA and ciprofloxacin showed a synergistic effect that caused a massive reduction in a *P*. *aeruginosa* biofilm. Overall, our results suggest the vast potential of M-HA as an antibacterial agent, which acts by specifically targeting a bacterial RNR enzyme.

## Introduction

Infectious diseases constitute a tenacious and major public health problem worldwide. For many years, antibiotic-resistant pathogens have been recognized as one of the primary threats to human survival, and some experts predict a return to the pre-antibiotic era. The emergence and increasing prevalence of bacterial strains that are resistant to available antibiotics urge the discovery of new therapeutic approaches [1]. An equally alarming decline has occurred in the research and development of new antibiotics to address the threat. Certain virulence factors have been shown to be potential targets for drug design and therapeutic intervention, and new insights are crucial for exploiting others [2, 3].

Bacterial DNA synthesis represents an attractive field for the discovery of new antibacterial targets because of remarkable differences from the eukaryotic system. During the course of infection, bacteria need to multiply inside the body, and they require active DNA synthesis to multiply. The key enzyme that provides the nucleotide precursors for DNA replication and repair are RiboNucleotide Reductases (RNRs). Three major classes of this enzyme are known (class I, II and III). Class I RNRs (subclasses Ia (*nrdAB*) and Ib (*nrdEF*)) carry a stable tyrosyl radical and are oxygen-dependent and thus, they only work under aerobic conditions; class II RNRs (*nrdJ*) require the vitamin B_12_ cofactor 5’-deoxyadenosylcobalamin and are oxygen-independent. Class III RNRs (*nrdD*, with their cognate *nrdG* activase) carry a stable glycyl radical, are oxygen-sensitive and only work under strict anaerobic conditions [4–8]. Eukaryotes only encode for one RNR class (Ia), but microorganisms have the ability to encode all possible RNR combinations [9, 10]. For this reason, RNRs could be considered a good antimicrobial target candidate to inhibit bacterial growth because they present substantial differences relative to their eukaryote counterparts [11, 12].

Several potential RNR inhibitors were reported, which included the following: free radical scavengers, iron chelators and substrate analogs [13–16]. Radical scavenger agents main mechanism of action is elicited through the inhibition of the RNR enzymes by scavenging the tyrosyl free radical (on the small class I subunits; NrdB and NrdF) that is required for the catalytic process [17]. These compounds have been shown to be useful for cancer treatment [18, 19]. One known family of radical scavenger compounds are derivatives from the hydroxylamine (HA) moiety, specifically hydroxyurea (HU), which is most commonly used for cancer treatment [18]. In fact, these compounds effectively inhibit the RNR of eukaryotic cells [19], reducing the possibility of using these drugs to treat bacterial infections without interfering in human RNR.

The modes by which different radical scavenger compounds interact in response to purified RNR enzyme were studied in our previous works [11, 12]. One compound, namely N-methyl-hydroxylamine (M-HA), was a highly active inhibitor of purified *Bacillus anthracis* RNR enzyme without interfering with the murine RNR enzyme. Although M-HA is promising as an antibacterial agent, its potential antimicrobial activity has not been evaluated.

In the present work, we aimed to explore the capacity of M-HA to inhibit the growth of clinically interesting Gram-positive (*Staphylococcus aureus*, *Streptococcus sanguinis*, *Streptococcus mutans* and *Mycobacterium*) and Gram-negative (*Pseudomonas aeruginosa* and *Burkholderia cenocepacia*) bacteria. Next, we investigated the effects of M-HA antibacterial activity on intracellular bacterial growth and biofilm-forming bacteria. Finally, the possible synergic activity of M-HA and other antimicrobials was evaluated.

## Materials and Methods

### Bacterial strains and mammalian cell line

*Streptococcus mutans* (ATCC 25175) and *Streptococcus sanguinis* (ATCC 10556) were grown in Todd-Hewitt broth (Oxoid) at 37°C. *Staphylococcus aureus* (ATCC 12600), *Pseudomonas aeruginosa* PAO1 (ATCC 15692) and *Burkholderia cenocepacia* J2315 (ATCC BAA-245) were grown in trypticase soy broth (TSB) or trypticase soy agar (TSA) (Sharlab, Barcelona, Spain) at 37°C. *Mycobacterium bovis* Bacillus Calmette-Guérin (BCG) substrain Connaught (ATCC 35745) was grown on Middlebrook 7H10 agar (Difco Laboratories, Surrey, UK) supplemented with 10% oleic-albumin-dextrose-catalase enrichment medium at 37°C for 2 weeks.

A murine macrophage J774A.1 cell line (DSMZ ACC 170) was maintained in Dulbecco’s Modified Eagle’s medium (DMEM) with L-glutamine (Gibco BRL, Grand Island, NY) supplemented with 10% heat-inactivated fetal bovine serum (FBS, Lonza Ltd., Switzerland) containing 100 U/ml penicillin G (Lab ERN, Barcelona, Spain) and 100 μg/ml streptomycin (Lab Reig Jofre, Barcelona, Spain) (complete medium) at 37°C in a humidified atmosphere with 5% CO_2_.

### Radical scavenger compounds

The hydroxylamine-bearing compounds used in this work were hydroxyurea (HU; *M =* 76.05 g/mol) (Sigma-Aldrich), hydroxylamine (HA; *M =* 33.03 g/mol) (Sigma Aldrich) and N-methyl-hydroxylamine (M-HA; *M =* 83.52 g/mol) (Acros Organics). Solutions were freshly prepared in PBS and filtered through a 0.22-μm pore-size filter (Millipore) before each experiment.

### Antibacterial susceptibility testing

To determine the survival of the different strains in the presence of different radical scavengers, each bacterial strain was grown in its specific medium to mid-log phase (A_550_ ≈ 0.5) and plated on solid plates supplemented with different concentrations of each compound.

In the case of BCG, colonies were scraped from Middlebrook 7H10 plates, resuspended in phosphate-buffered saline (PBS), slightly vortexed with glass beads to dissolve clumps, and allowed to settle for 30 minutes. The supernatant was diluted in PBS and adjusted to 1.0 McFarland standard. Serial dilutions were then plated on solid plates containing freshly prepared compounds at the indicated concentrations. Colony-forming units (cfu) were counted after growing.

Inhibitory concentration 50% (MIC_50_) was defined as the compound concentration that reduced bacterial growth (cfu) by 50%, and MIC_100_ was defined as the lowest concentration of drug that visibly inhibited bacterial growth by 100%.

### Determining mammalian cytotoxicity

Murine J774 macrophages (6x10^4^ per well) were seeded onto 48-well tissue culture plates in complete medium without antibiotics in the presence of different doses of HU, HA and M-HA, or left untreated. After 24, 72 and 120 h of exposure to the different compounds, culture supernatants were removed and cell viability was assessed by using a 3-[4,5-dimethylthiazol-2-yl]-2,5-diphenyltetrazolium bromide (MTT) colorimetric assay (Sigma Aldrich) (20). Absorbance was measured at 550 nm with an ELISA reader (Infinite M200 Microplate Reader, Tecan). The results were expressed as a percentage of cell survival relative to untreated cells. Each experiment was repeated at least three times.

In another set of experiments, cells were washed at 24 hours after adding the compounds for the first time, and new, freshly made compounds were added. Cell viability was measured each 24 hours as described above.

The 50% cytotoxicity inhibitory concentration (CC_50_) of each drug was determined from dose-response curves by using Graph Pad Prism v6. The selectivity index (SI) (SI = CC_50_/MIC_50_) was calculated on the basis of the CC_50_ and MIC_50_ values as determined after 24 h of exposure.

### The anti-BCG intracellular activity of the different compounds

For the infection experiments, BCG suspensions were adjusted to a 1.0 McFarland standard and centrifuged at 2000 g for 10 minutes. The bacterial pellets were re-suspended in complete medium without antibiotics and were further subjected to three consecutive 30 second pulses (45 W) in an ultrasonic water bath to obtain a predominantly single bacterial cell suspension.

Murine J774 macrophages (3x10^4^ per well) were seeded onto 48-well plates in complete medium without antibiotics. Twenty-four hours later, they were infected with BCG at a multiplicity of infection (MOI) of 10 for three hours as previously described [20]. The MOI was confirmed by plating serial dilutions of the inoculum on solid media. After three hours, cells were washed to remove extracellular bacteria and incubated with fresh complete medium plus different doses of HU, HA, and M-HA, at 37°C in a 5% CO_2_ atmosphere. All infections were performed in triplicate. The cell culture supernatants were removed, macrophages were lysed, and bacterial counts were determined by plating serial dilutions on Middlebrook 7H10 plates at different time points after infection (24, 72, and 120 h) [20]. Non-infected control cultures were always included and all experiments were repeated at least three times.

### Cytokine analysis and nitric oxide (NO) production

Cell culture supernatants were collected at different time points as indicated above in both experiment types for macrophage cell viability and intracellular BCG susceptibility to the compounds. Interleukin (IL)-10, IL-12 and tumor necrosis factor (TNF)-α levels were determined by using commercially available enzyme-linked immunosorbent assays (ELISA) (IL-10 and IL-12p40 from Mabtech AB, Nacka Strand, Sweden; and TNF-α from R&D Systems Inc., Minneapolis, MN, USA) according to the manufacturer’s instructions. All samples were assayed in duplicate.

The NO production was assessed by measuring nitrite concentrations with the Griess reaction (Sigma).

### Viability test analysis

An overnight culture of *P*. *aeruginosa* PAO1 was diluted in fresh LB medium and grown to the beginning of exponential phase (A_550_ ∼ 0.3) to which different concentrations of radical scavenger compounds were added. After 3 or 24 hours of incubation at 37°C, the cells were stained by using the LIVE/DEAD BactLight viability kit (Life Technologies) for 15 minutes at room temperature in the dark. Fluorescent bacteria were visualized with a Nikon E600 microscope (Nikon) coupled with a DP72 Olympus camera.

### Viable cell counts in biofilm inhibition after radical scavenger treatment

To investigate the anti-biofilm activity of the different radical scavengers alone or in combination with ciprofloxacin, *P*. *aeruginosa* PAO1 biofilms were grown on microtiter plates and a previously described protocol was followed [21]. An overnight-grown culture of *P*. *aeruginosa* in TSB was diluted 1:100 in sterile TSB medium supplemented with 0.2% glucose and added to a 96-well microtiter plate with pegs (Nunc-TSP, Thermo Scientific) (200-μl each well). After 24–48 h of incubation at 37°C in a humidified chamber, the culture supernatant was discarded and the pegs were washed three times with sterile PBS to remove non-adherent cells. After being rinsed, the biofilms were treated with different radical scavenger concentrations alone or in combination with ciprofloxacin. After 24 h of treatment, the pegs were transferred to a new plate that had been rinsed with PBS. Adherent bacteria were first fixed with 200 μl of methanol for 10 min and then stained with crystal violet (1%) for 10 min. Excess crystal violet was washed gently with water and the pegs were dried in air for 5 min. The dye that was bound to the cells was dissolved with 150 μl of ethanol 95%, centrifuged at 2000 rpm for 10 min, and read at 570 nm with a microplate reader (Infinite M200).

### Biofilm culture in flow cell system and confocal microscopy analysis

To prepare biofilms developed under continuous flow, *P*. *aeruginosa* cells (5x10^5^ cfu/ml) were cultured in LB medium at 25°C in flow chambers with channel dimension of 1x4x40 mm as described previously [22]. After 96 h of culture, formed biofilms were treated with 40 μg/ml of HA, HU or M-HA, and LB medium was used alone in the control sample. After 24 h of treatment at 25°C, biofilms were stained with 5 μM SYTO 9 at room temperature in the dark for 30 min, according to the specifications of the LIVE/DEAD BacLight Bacterial Viability kit (Molecular Probes, Invitrogen).

Confocal scanning laser microscopy of the biofilms was performed with a Leica TCS-SP5 confocal scanning laser microscope (Leica Microsystems, Wetzlar, Germany), with an excitation wavelength of 477 for SYTO9. To measure biofilm thickness, sections were scanned and Z-stacks were acquired at z step-size of 0.388 μm. Field size was 456 μm x 456 μm at 20X magnification. Microscope images were further processed with ImageJ analysis software (National Institute of Health, USA) and COMSTAT 2 software, specific for biofilm quantitative analysis [23].

### Statistical analyses

Data were presented as the means ± standard deviation (SD). The statistical significance of differences between cytokine levels and BCG growth inhibition using the different radical scavenger compounds was assessed by using Student’s t-tests (SigmaStat, SPSS, Chicago, IL). Differences were considered significant when *P* < 0.05. All statistical procedures were performed with SPSS 15.0 software (SPSS Inc., Chicago, IL).

## Results

### M-HA showed greater antibacterial activity than HU and HA radical scavengers

The antibacterial activity of three radical scavengers (HU, HA and M-HA) was evaluated against four Gram-positive bacteria (*S*. *aureus*, *S*. *mutans*, *S*. *sanguinis* and *M*. *bovis* BCG) and two Gram-negative bacteria (*P*. *aeruginosa* and *B*. *cenocepacia)*. As shown in Table 1, the HU compound exhibited moderate activity against *S*. *aureus*, *S*. *mutans* and *S*. *sanguinis* (200–330 μg/mL) and high growth inhibitory activity against *P*. *aeruginosa*, *M*. *bovis* and *B*. *cenocepacia* (7.6 to 13 μg/mL). HA showed better growth inhibitory activity relative to HU in all tested bacteria (2 to 52 μg/mL). M-HA was highly active in *M*. *bovis* BCG and *P*. *aeruginosa* cultures, with a 1.5 to 4.5-fold lower MIC_50_ than HU and HA. In BCG cultures, 7.5 to 43 times lower concentrations of M-HA (MIC_50_ = 1.9 μg/mL) were needed to obtain the same results as the other bacteria cultures (from 14.2 μg/mL for *B*. *cenocepacia* to 81.9 μg/mL for *S*. *sanguinis*) (Table 1).

**Table 1:** The antibacterial activity of HU, HA and M-HA against Gram-positive and Gram-negative bacteria.

	HU μg/ml				HA μg/ml				M-HA μg/ml			
	MIC_50_	MIC_100_	CC_50_	SI	MIC_50_	MIC_100_	CC_50_	SI	MIC_50_	MIC_100_	CC_50_	SI
***S*. *aureus***	279.9	>380	25.9	0.09	28.4	<132.2	12.9	0.45	58.5	<334.1	350.8	6
***S*. *mutans***	205.3	>1140	25.9	0.13	16.5	<231.4	12.9	0.78	77.7	<584.7	350.8	4.5
***S*. *sanguinis***	336.9	>1140	25.9	0.08	51.5	<231.4	12.9	0.25	81.9	<417.6	350.8	4.3
***M*. *bovis BCG***	8.36	>380	25.9	3.1	2.6	<16.5	12.9	4.96	1.9	<41.8	350.8	184.6
***P*. *aeruginosa***	7.6	<41.8	25.9	3.4	8.3	<66.1	12.9	1.55	6.7	<108.6	350.8	52.4
***B*. *cenocepacia***	12.9	>129.3	25.9	2	2.6	>66.1	12.9	4.96	14.2	>142	350.8	24.7

The growth inhibition of different bacteria after culturing them in HU-, HA-, or M-HA-supplemented media. The data are representative of one of at least three independent experiments. HU, hydroxyurea; HA, hydroxylamine; M-HA, methyl-hydroxylamine; MIC_50_, 50% inhibitory concentration; MIC_100_, 100% inhibitory concentration; CC_50_, 50% cytotoxicity inhibitory concentration; and SI, selectivity index (SI = CC_50_/MIC_50_).

**Fig 1:**
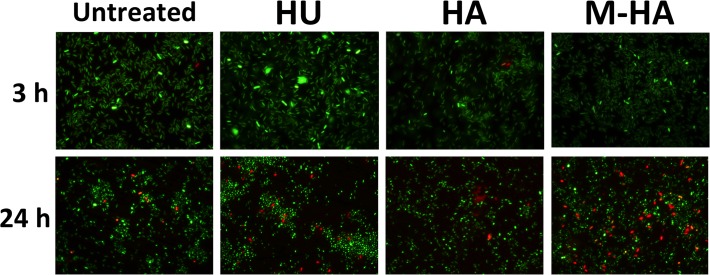
*P*. *aeruginosa* viability according to the LIVE/DEAD assay after treating with HU, HA, and M-HA. The bacteria were grown with HU (7.6 μg/ml), HA (8.3 μg/ml), or M-HA (6.7 μg/ml) for 3 and 24 hours and stained with LIVE/DEAD assay. Live cells were green (SYTO 9 dye) and dead cells were red (propidium iodide dye) under a fluorescent microscope. Magnification, x 1000. HU, hydroxyurea; HA, hydroxylamine; and M-HA, methyl-hydroxylamine.

To investigate the mechanisms through which M-HA inhibits bacteria growth, we specifically stained bacteria with the Live/Dead BactLight bacterial viability assay (Invitrogen). As shown in Fig. 1, the different radical scavengers (at a MIC_50_ concentration) did not apparently modify the bacterial membrane integrity after 3 hours of treatment because all of them were stained green. After 24 h of treatment, the proportion of non-viable cells (red cells) increased, especially when treated with M-HA.

### M-HA does not affect eukaryotic cell growth

**Fig 2:**
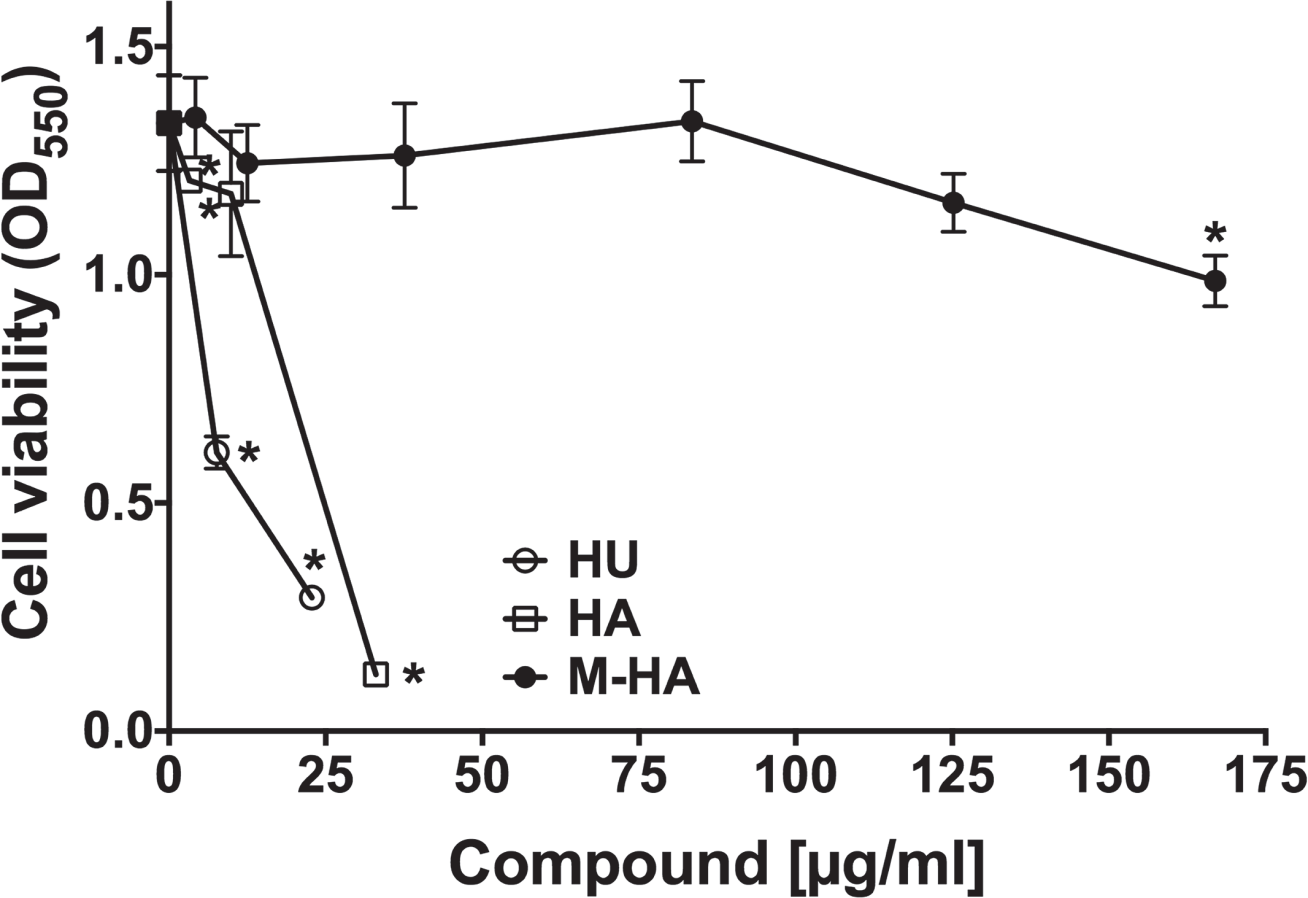
Dose-response curves for HU, HA and M-HA treatments of murine macrophages. The level of growth alteration for J774 macrophages that were treated with different doses of radical scavenger compounds at 72 hours post-treatment. Cell viability was measured by using an MTT assay. Values represent the means ± standard deviation (SD) of triplicate cultures. The data are representative of one of at least three independent experiments. (*, *P* < 0.05 *vs*. non-treated cells). HU, hydroxyurea; HA, hydroxylamine; and M-HA, methyl-hydroxylamine.

The antiproliferative activity of the three radical scavengers was evaluated against murine J744 macrophages by MTT staining. As expected, HU and HA interfere with macrophage proliferation even when low concentrations are used (Fig. 2). Only the lowest dose of HA (10 μg/ml) permits macrophage growth. By contrast, concentrations of up to 250 μg/ml M-HA do not inhibit macrophage proliferation (Fig. 2). The same results were obtained when culture medium plus radical scavenger compounds were renewed every 24 hours (data not shown), and even if the cells were treated for up to 120 hours (data not shown). As shown in Table 1, a cytotoxic concentration (CC_50_) is observed for M-HA when doses higher than 250 μg/mL were used.

The selectivity index (SI) was calculated on the basis of the MIC_50_ and CC_50_ values that were determined after 24 h of exposure (Table 1). High SI values were obtained for M-HA in *Mycobacterium* and *Pseudomonas* growth inhibition (SI = 182.6 for *M*. *bovis* and 52.5 for *P*. *aeruginosa*), which were much higher than the SI obtained with HA or HU (SI from 0.08 to 4.9).

### M-HA shows intracellular antimycobacterial activity

**Fig 3:**
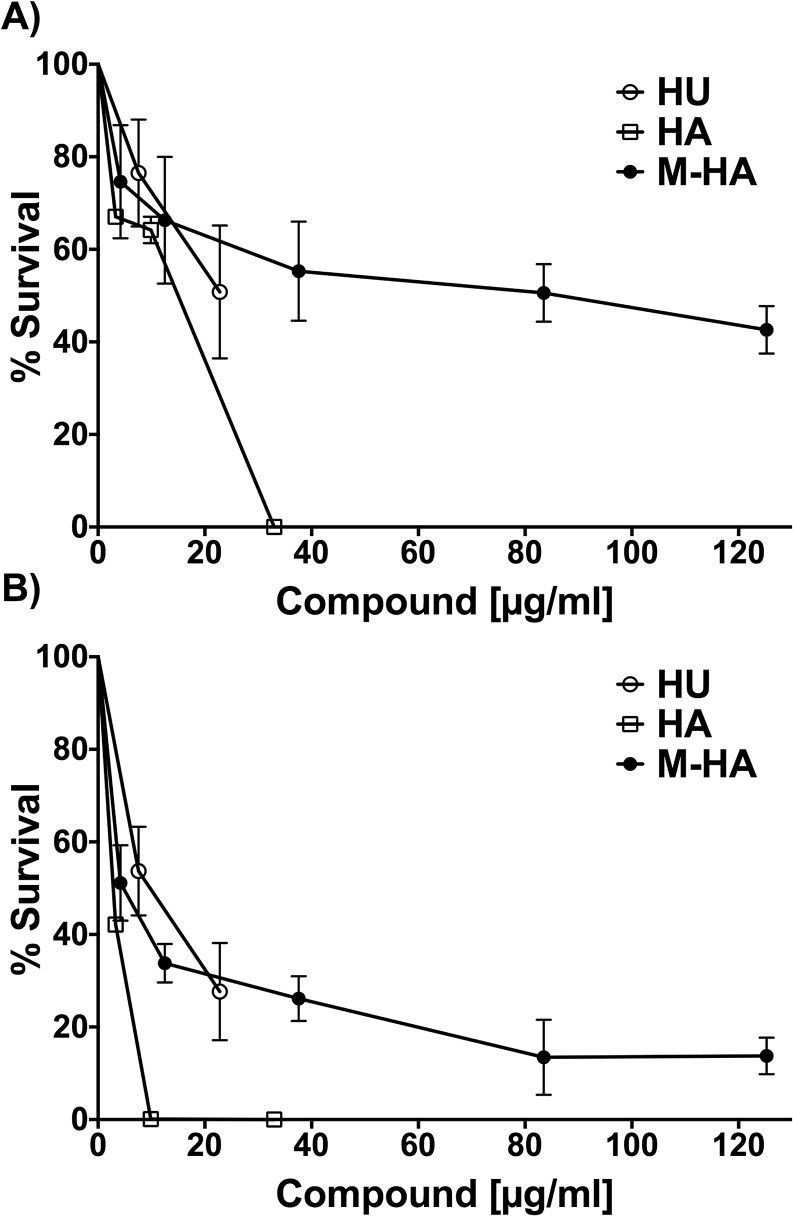
The intracellular BCG growth inhibition of macrophages treated with different doses of HU, HA and M-HA. The effects of different doses of radical scavenger compounds on the intracellular viability of BCG at 72 hours post-infection. A) Compounds were added 3 hours after infection B) Compounds were renewed every 24 hours after infection. The results are expressed as the means ± standard deviation (SD) of triplicate wells in percentages of inhibition with respect to non-treated cells. The data are representative of one of at least two independent experiments where statistically significant differences between treated and untreated cells (*P* < 0.05) for all compounds and concentrations. HU, hydroxyurea; HA, hydroxylamine; and M-HA, methyl-hydroxylamine.

In view of the results shown in Table 1, M-HA seems to be a promising antimycobacterial candidate because mycobacteria are the intracellular pathogens for which we aimed to demonstrate activity in infected macrophages. As shown in Fig. 3A, *M*. *bovis* BCG viability was diminished when infected macrophages were incubated in the presence of the different radical scavenger compounds related to untreated wells. At 72 hours post-infection, HU triggers a BCG growth inhibition of approximately 50%, but only HA treatments exhibited better inhibition values when the dose was greater than 35 μg/ml. As explained before, this finding also corresponds to a concomitantly drastic reduction in macrophage viability (see Table 1, CC and Fig. 2). By contrast, M-HA showed enhanced intracellular *M*. *bovis* BCG growth inhibition in comparison with that of HU or HA at a range of concentrations that were not toxic for eukaryotic cells between 74.6% BCG survival at 8 mg/ml and 42.6% at 125 mg/ml (Fig. 3A). Moreover, the M-HA intracellular antimycobacterial activity improved when the culture medium was changed every 24 hours by adding freshly prepared compounds, and values of up to 13% BCG survival were reached (Fig. 3B). In absolute numbers, this finding represents up to a one log reduction, i.e., at 72 hours post-infection, an M-HA concentration of 82 mg/ml was reduced from 6.3x10^4^ (untreated wells) to 8.4x10^3^ viable BCG cells (cfu). Similar values were observed in both cases (replacing or not replacing the compounds every 24 hours) at 120 hours post-infection (data not shown).

### Increased TNF production by M-HA

**Fig 4:**
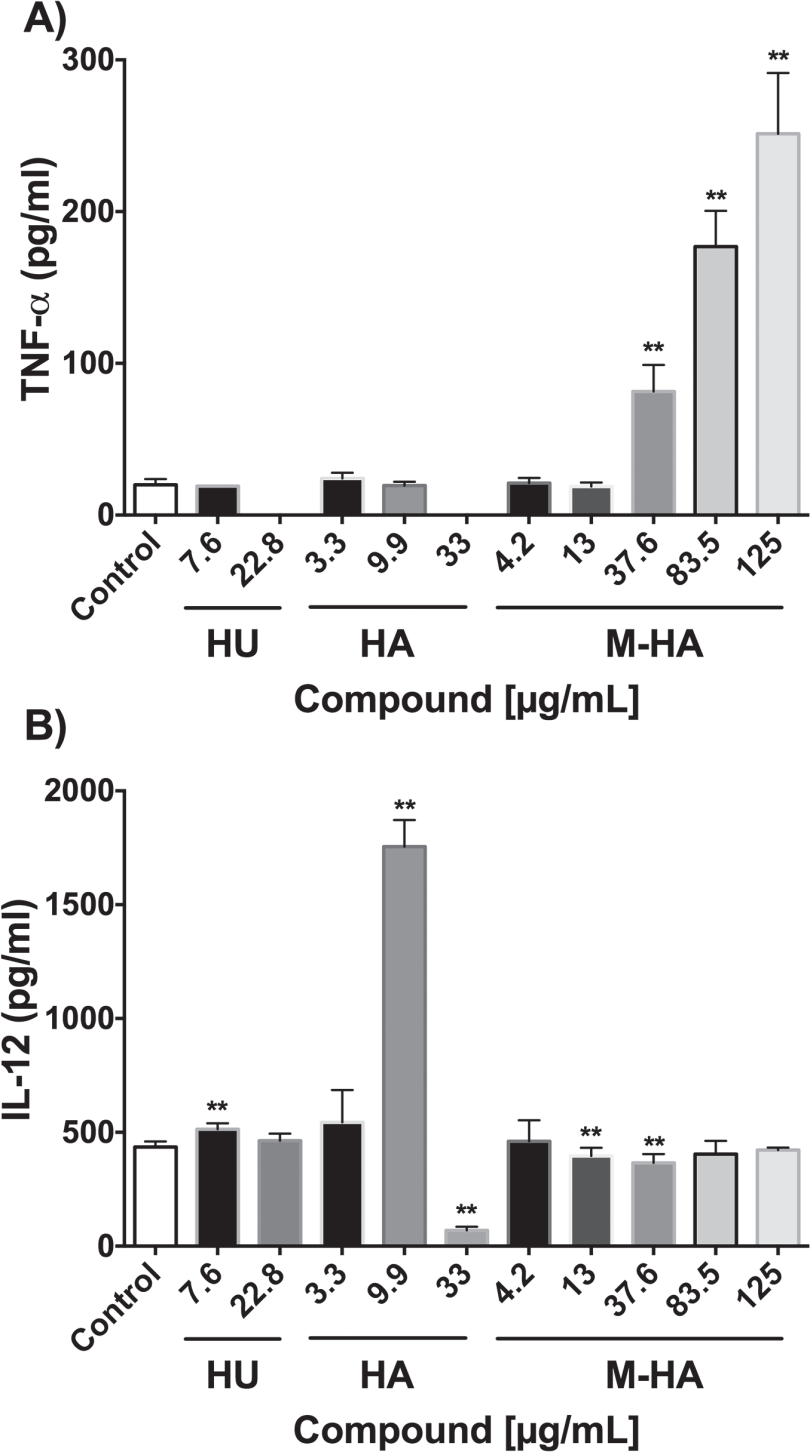
TNF-α and IL-12 production as triggered by BCG-infected macrophages that were treated with different doses of HU, HA and M-HA. J774 macrophages were infected with BCG and treated with different concentrations of radical scavenger compounds, and TNF-α and IL-12 levels were measured 24 hours post-infection. The results represent the means ± SD of triplicate preparations with one representative of two independent experiments. A Mann-Whitney test was performed (*, *P* < 0.01; versus non-treated macrophages (control)). HU, hydroxyurea; HA, hydroxylamine; and M-HA, methyl-hydroxylamine.

We investigated the production of two bactericidal products that are able to kill intracellular BCG, namely TNF-α and NO [24], when the macrophages were infected with BCG and treated with the different radical scavenger compounds. As shown in Fig. 4, M-HA-treated macrophages produce higher TNF-α values at 24 hours post-infection than untreated cells (Fig. 4A). The highest amount of cytokine production was observed when cells were treated with high concentrations of M-HA (Fig. 4A). The amount of cytokine production did not increase after longer periods of incubation (72 or 120 hours after infection) (data not shown). When TNF-α production was evaluated in non-infected cultures, similar values were obtained in radical scavenger-treated and non-treated macrophages (data not shown).

Low but detectable amounts of NO production between 1 and 3 μM of NO were found in both BCG-infected radical scavenger-treated and untreated macrophages. No significant differences were observed between HA, HU and M-HA-treated macrophages. These data are consistent with previous data from [25].

When the production of IL-10 and IL-12 was studied in BCG-infected macrophages treated with the different radical scavengers, the results differ between cytokines and treatments. While IL-10 production was not detected in any case (data not shown), IL-12 production was significantly increased in HA-treated macrophages in a dose-dependent manner (Fig. 4B). At the highest HA concentration, however, IL-12 production dramatically diminished, probably due to the reduced presence of viable macrophages (Fig. 2).

### M-HA inhibits *P*. *aeruginosa* biofilm formation

**Fig 5:**
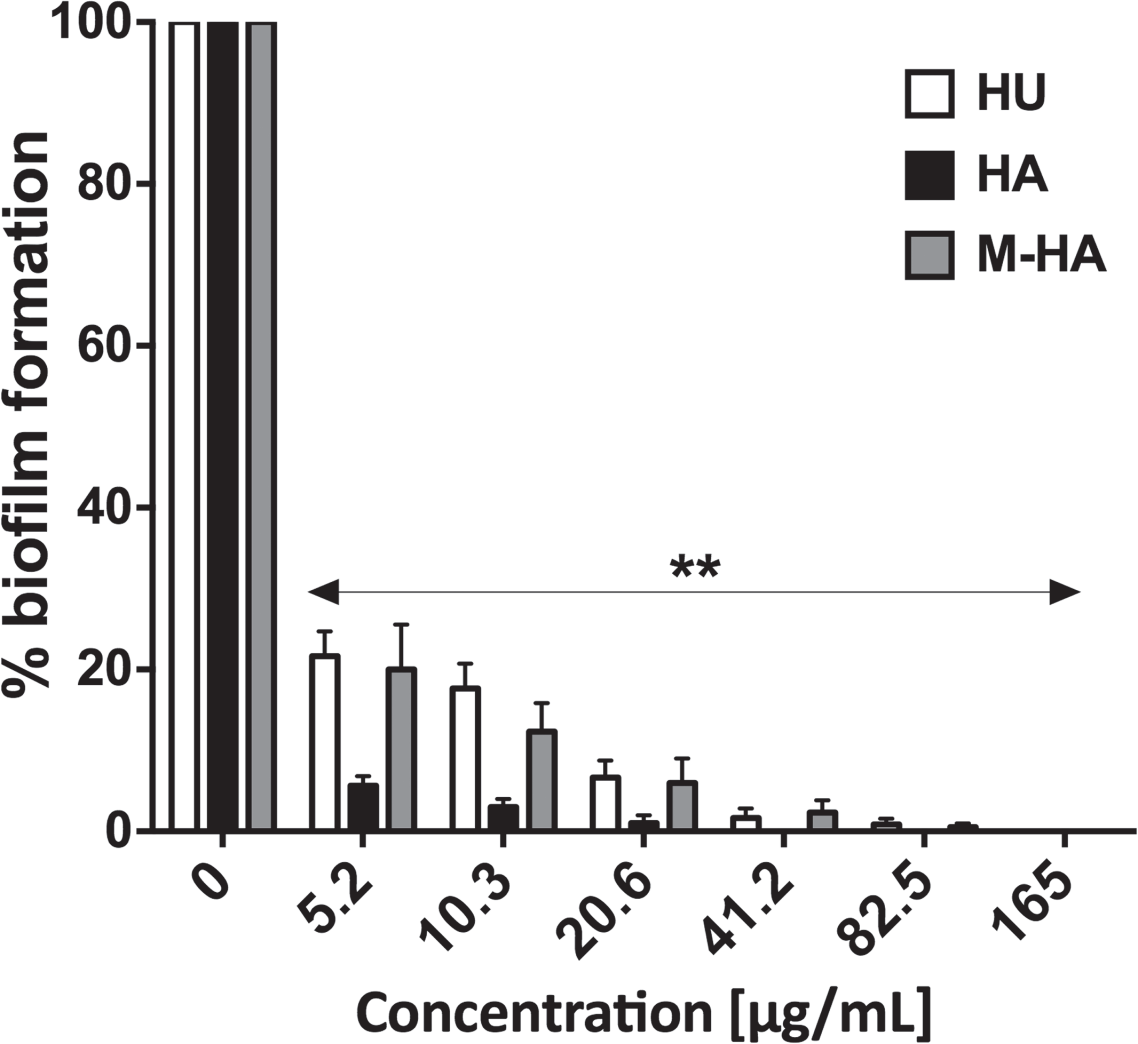
The inhibition of biofilm formation by different doses of HU, HA and M-HA. The biofilm formation of *P*. *aeruginosa* PAO1 was quantified as the absorbance of crystal violet stain (A_570_ nm) after being cultured for 24 h in 96-well plates in the presence and absence of the radical scavenger compounds. The values represent the percentages of biofilm biomass production. The results are expressed as the means ± SD of five replicates from one representative of three independent experiments. A Student's t-test was performed (**, *P* < 0.01; versus non-treated biofilms). HU, hydroxyurea; HA, hydroxylamine; and M-HA, methyl-hydroxylamine.

As explained previously, good M-HA antibacterial activity was also observed against *P*. *aeruginosa* (SI = 52.4). Thus, we further investigated the capacity of M-HA in reducing *P*. *aeruginosa* biofilms because it is one of the most important forms of persistent bacteria and is a characteristic of chronic *P*. *aeruginosa* infections. We used the quantitative microtiter plate method to determine the effect of the different hydroxylamine derivative compounds on biofilm formation. A dose-effect concentration was observed for each compound (Fig. 5). At 20.6 μg/ml HA and 82.5 μg/mL HU and M-HA, *P*. *aeruginosa* growth was completely arrested and no biofilm was formed (Fig. 5).

### The M-HA effect on a preformed *P*. *aeruginosa* biofilm

**Fig 6:**
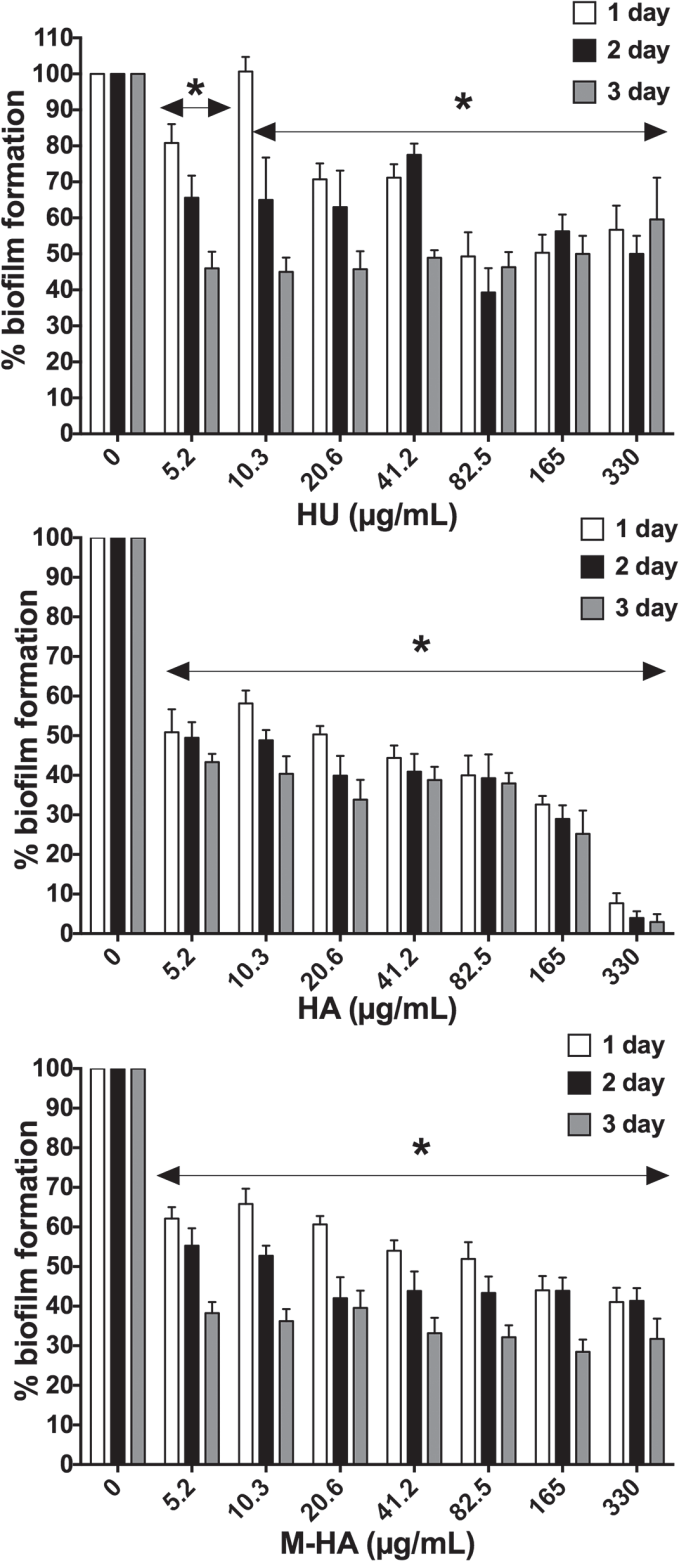
Disassembling the existing *P*. *aeruginosa* biofilms by adding HU, HA and M-HA. *P*. *aeruginosa* bacteria were allowed to form biofilms in peg plates for 24 h, the medium was removed and fresh medium with different concentrations of radical scavenger compounds were changed every 24 hours over three days (Days 1, 2 and 3). The percentage of biofilm biomass production is represented for each day. The results are the means ± SD of three-five replicates from one representative of two independent experiments. A Student's t-test was performed (*, *P* < 0.05; versus non-treated biofilms). HU, hydroxyurea; HA, hydroxylamine; and M-HA, methyl- hydroxylamine.

**Fig 7:**
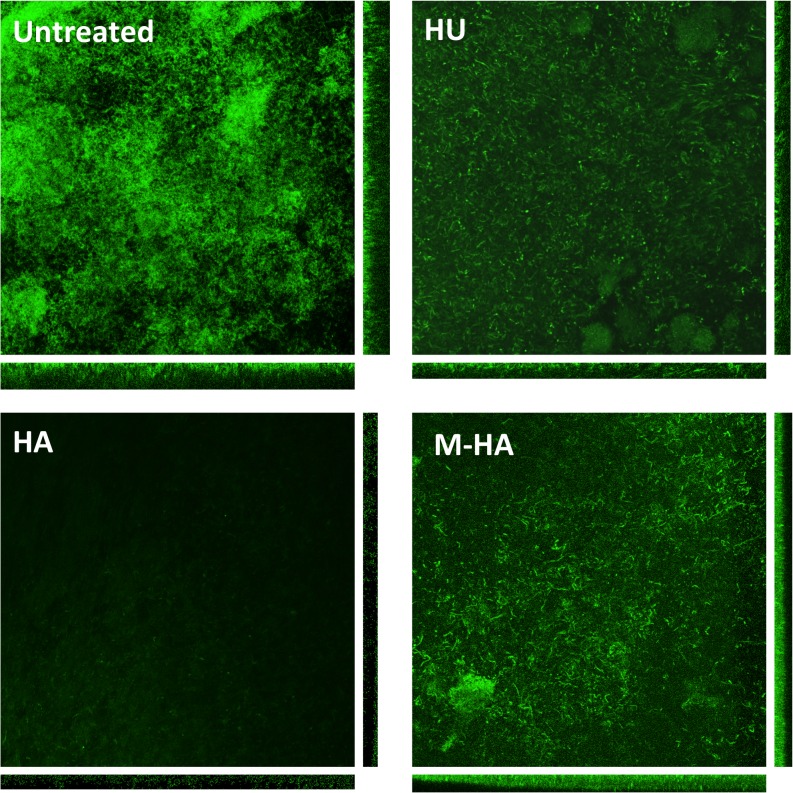
Confocal microscopy of *P*. *aeruginosa* biofilms grown on flow cell. The formed biofilms were treated for 24 h with HU, HA and M-HA at 40 **μ**g/ml final concentration. Each panel shows the maximum Z-projection and the orthogonal views for each stack.

Once a biofilm has been established, the cells are extremely resistant against all types of antibiotics and detergents and it is often challenging to remove. The effects of the different radical scavengers on existing *P*. *aeruginosa* biofilms were initially assessed using crystal violet-based biomass staining assay. As Fig. 6 shows, all compounds reduce the amount of biofilm formed, reaching values of approximately 55%, 90% and 70% reduction from HU, HA, and M-HA, respectively, at the highest tested concentrations and three days of treatment. When employing M-HA concentrations that did not affect eukaryotic cell viability (up to 165 mg/ml), increasing amounts of compound diminished the established biofilms, indicating that the cells in an existing biofilm can be removed or disaggregated. Moreover, the highest biofilm reductions under M-HA treatment were found after three days of treatment, with 20% less remaining biofilm compared with the first day of treatment (Fig. 6). Further, confocal scanning laser microscopy of a biofilm grown in a flow cell chamber in the presence of the different radical scavengers showed significantly reduced biomass and average thickness of the treated samples (HA, HU and M-HA) compared to the untreated sample (Table 2 and Fig. 7).

**Table 2:** Biofilm parameters of wild-type *P*. *aeruginosa* treated with 40 μg/ml HA, HU and M-HA.

	Live cells (green)	
	Biomass (μm^3^/μm^2^)	Averange thickness (μm)
**Untreated**	28.9 ±4.7	40.2±3.5
**HU (40 μg/ml)**	11.9±4.5*	24.2±8.2*
**HA (40 μg/ml)**	10.7±3.2*	19.4±2.5*
**M-HA (40 μg/ml)**	20.5±5.9*	30.6±2.9*

Biomass values indicate the amount of living cells inside the biofilm. Values represent the mean ± SD of three independent experiments. Asterisk denotes significant differences compared to non-treated biofilm (p<0.05, Student’s *t*-test).

### Synergic effects on biofilm reduction by ciprofloxacin plus M-HA

**Fig 8:**
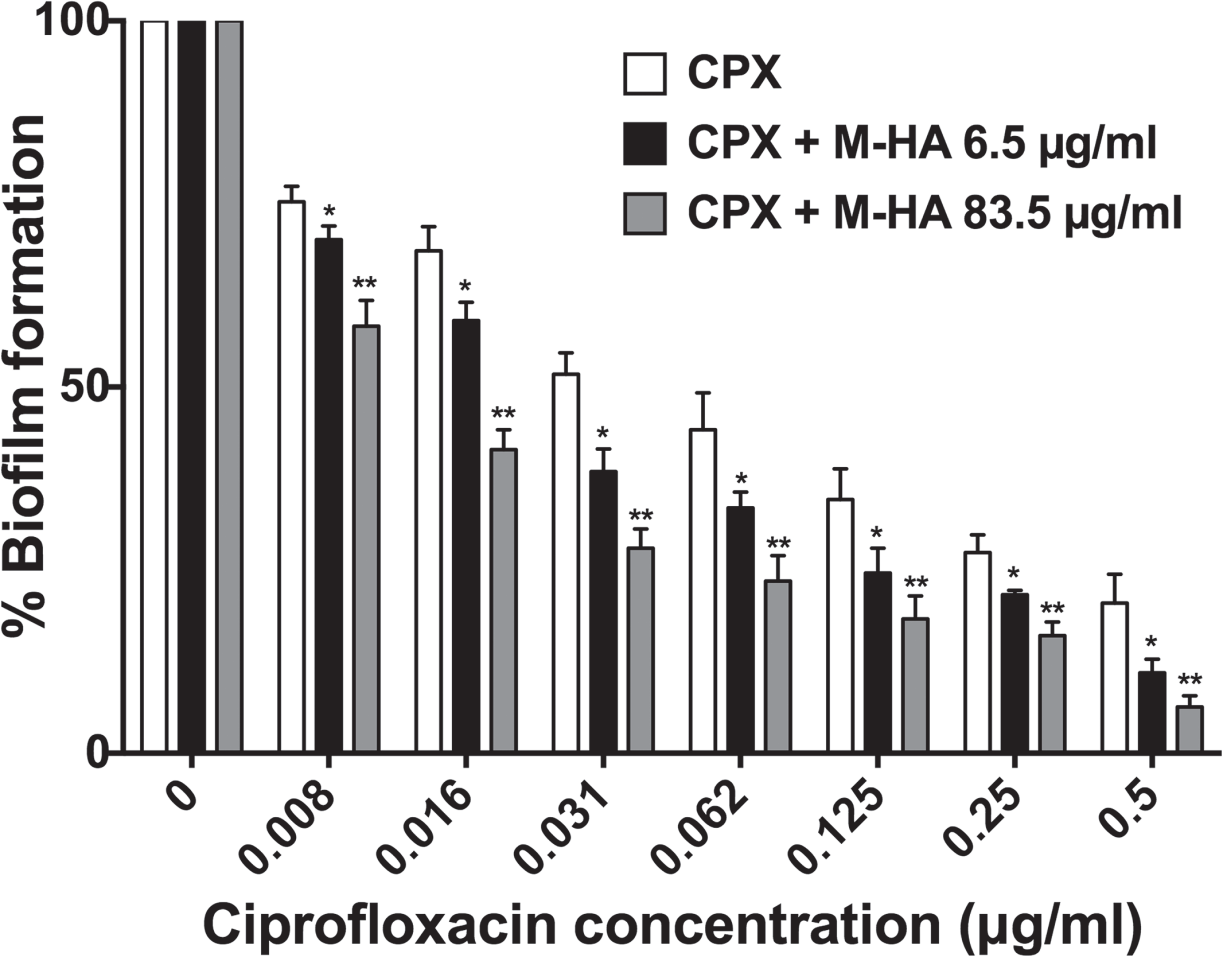
The synergistic effect of ciprofloxacin and M-HA on the reduction in *P*. *aeruginosa* biofilm formation. *P*. *aeruginosa* bacteria were allowed to form biofilms in peg plates for 24 h, the medium was removed and fresh medium with different concentrations of ciprofloxacin with/without M-HA were added. Biofilm formation (crystal violet stain) was evaluated 24 hours later. The biofilm biomass production percentage is represented here. The results are the means ± SD of three-five replicates from one representative of two independent experiments. A Student's t-test was performed (*, *P* < 0.05; **, *P* < 0.005 versus ciprofloxacin treated biofilms). CPX, ciprofloxacin; and M-HA, methyl-hydroxylamine.

The capacity to remove a pre-existing biofilm was evaluated when treating with ciprofloxacin in combination with M-HA. Ciprofloxacin alone showed a dose-dependent effect on *P*. *aeruginosa* biofilm reduction (Fig. 8). However, in comparison with treatments that employed ciprofloxacin alone, the combined use of ciprofloxacin and M-HA was more efficient for removing pre-existing biofilm, yielding 50% reduction values at ciprofloxacin concentrations of 0.016 or 0.008 **μ**g/ml, and 6.6 or 86 **μ**g/m of M-HA, respectively, was added. (for 5 to 8 times lower concentration than ciprofloxacin alone to have the same effect) (Fig. 8).

## Discussion

The clinical use of RNR inhibitors has a history of several decades, and this history has demonstrated that RNR inhibitors have antitumor activity alone or in combination with other drugs [13, 18, 19]. Among RNR inhibitors, some radical scavenger compounds derived from hydroxylamines such as HU have been used for some types of cancer treatment [18]. Both HA and HU dramatically affect eukaryotic viability, and thus little interest has been aroused in studying these compounds as antimicrobial agents. It remains an important goal to develop a novel HA derivative with low toxicity and improved cytostatic action especially for treating bacterial infections. We previously demonstrated the capacity of the HA derivative M-HA in inhibiting RNR enzymatic activity in *B*. *anthracis* [11], although its potential as an antimicrobial drug has not been investigated. When exploring the role of M-HA in inhibiting the growth of a wide range of pathogenic Gram-positive or Gram-negative bacteria during a comparison of HA and HU, the intracellular bacterial growth and the formation of biofilms was evaluated.

Our results show that HA and M-HA are more efficacious that HU as antimicrobial agents for both Gram-positive and Gram-negative bacteria (Table 1). For instance, *P*. *aeruginosa* and *M*. *tuberculosis* use an iron containing class I RNR (class Ib) [26, 27] while *B*. *anthracis* clearly uses a manganese class I RNR (Class Ib) [28]. Our results suggest that M-HA is an active inhibitor for both iron and manganese forms of RNR.

When eukaryotic cytotoxicity was evaluated, HU and HA exerted high toxicity in murine macrophage cells (CC_50_ of 25.8 and 12.9 **μ**g/ml, respectively) as expected (Fig. 2). Our results are consistent with previous results in which HU and resveratrol (a radical scavenger aromatic compound that is not derived from HA) were efficacious against pathogenic bacteria (*P*. *aeruginosa*, *Propionibacterium acnes*, *S*. *aureus*, and *Enterococcus faecalis*) but had high toxicity in eukaryotic cells as well [29, 30]. However, M-HA showed a highly reduced toxic effect on macrophage culture (CC_50_ of 351 **μ**g/mL), corroborating the low toxicity of M-HA that was also found against human lung fibroblasts [31]. Thus, our first results confirm the promising role of M-HA as an antimicrobial agent.

Because of their small sizes, these compounds would easily cross the cell wall and membrane and exert their antimicrobial activity directly through RNR enzyme inhibition. In previous works, we determined that M-HA specifically inactivates the bacterial enzymatic activity of the essential RNR enzyme by quenching the tyrosyl radical that is necessary for enzymatic activation, thus making it unable to form dNTPs and blocking DNA synthesis [11]. The exact mode of M-HA is not completely understood, but some authors hypothesize that this molecule can interact directly in the places where the tyrosyl radical is formed because of its small size [32]. In terms of HU, which is a bigger molecule than M-HA, it seems that the interaction does not occur directly where the tyrosyl radical is generated but is more directed at interrupting the catalytic electron transfer pathway of the small subunit at the interface between the interaction between the small (α) and large RNR (β) subunits [33–35]. This finding could explain why M-HA was approximately several orders of magnitude more effective at inhibiting bacterial RNR than HU. Confirmation was established with the use of HA, the smallest HA, with the highest antimicrobial activity that also presents the highest toxicity because this molecule surely interacts with and can reach the tyrosyl radical site easily in both prokaryotic and eukaryotic RNR. Another issue is to understand the precise mechanism of action for the bacterial killing of radical scavengers after blocking the RNR enzyme. In *E*. *coli* [36] HU has been recently shown to cause increased superoxide production, and together with increased iron uptake, this increase fuels the formation of hydroxyl radicals that contribute to HU-induced cell death. Based on the HU analogy, we believe that the M-HA mode of action or bacterial killing might be similar.

Interestingly, M-HA has the highest therapeutic index for cytotoxicity (SI) for its inhibition of *M*. *bovis* BCG (182.6 **μ**g/ml), which is 3.5 times that of *P*. *aeruginosa* (52.5 **μ**g/ml). Because of the global relevance of these two agents, we should search for the possible role of M-HA in treating these agents.

To measure the potential growth inhibitory capacity of these compounds against mycobacteria, we selected BCG as a model in murine macrophages. Although there are significant differences between *M*. *tuberculosis* and BCG, they are closer genetically, and previous studies have demonstrated the validity of BCG for the in vitro evaluation of drug candidates against tuberculosis [37, 38]. In our case, a comparison of the BCG RNR primary protein structure showed 100% shared identity with that of the *M*. *tuberculosis* RNR protein, the causative agent of tuberculosis (see S1 Fig.). This finding indicates a possible identical mode of action for M-HA on the *M*. *tuberculosis* RNR. BCG intracellular kinetics in J774 macrophages has been very well characterized [25, 39]. Initially, J774 macrophages ingest mycobacteria and internalize BCG cells into phagosomes. An initial killing is observed during the first three days of infection, and then a stable level of viable BCG can be observed in J774 macrophages. Despite the fact that BCG does not grow as exponentially inside macrophages as *M*. *tuberculosis* does, BCG is continuously growing and being killed by macrophages [25]. This finding permits the use of this model for evaluating new drug candidates with the capacity to interfere in mycobacterial DNA synthesis.

Our results showed that up to 85% of mycobacterial growth inhibition by M-HA occurs at 72 hours post-infection (Fig. 3). In view of the impressive results obtained here, we aimed to go further in understanding the mechanism. As expected [40], our results showed that BCG-infected macrophages do not induce NO production. Treatment with HA-derivatives did not significantly modify these results. However, enhanced TNF-α production, which is another killing mechanism of macrophages, is observed in M-HA-treated macrophages. The increased production of TNF-α in BCG-infected M-HA-treated macrophages could be explained by different reasons. On the one hand, BCG-infected J774 macrophages release exosomes that contain mycobacterial antigens such as a 19 kDa antigen or phosphatidyl inositol mannosides known to induce pro-inflammatory cytokines such as TFN-α by exerting bystander effects on other cells [41]. The M-HA treatment could induce a higher production of these exosomes than non-treated macrophages. On the other hand, M-HA could directly induce TNF-α production in BCG-infected macrophages. Two reasons led us to support this last option. First, TNF-α production is observed as early as 24 hours post-infection (Fig. 4), whereas the exosome released from BCG-infected J774 macrophages is primarily detected between 48 and 72 hours post-infection [41]. Second, an M-HA dose-dependent response is observed in TNF-α production, leading us to favor the second hypothesis. The fact that TNF production levels in non-infected M-HA-treated macrophages were lower than those detected in M-HA-treated BCG-infected macrophages indicates that a BCG-infection must be related to the capacity of M-HA to induce TNF-α production, as was previously described for other drugs [42, 43]. Nevertheless, we cannot rule out other possible mechanisms, such as the influence of such radical scavengers in other routes related to cytokine production. Supporting this idea, significant IL-12 levels were only detected in BCG-infected HA-treated macrophages. Although in vitro and in vivo data indicated that HU or HA treatment triggers chemokines and/or cytokines production [44–48], the mechanism by which these compounds interfere with cytokine-mediated signaling is also unclear. TNF-α and IL-12 are critical cytokines in the control of mycobacterial infections [49]. Synergically with IFN-γ, the compounds activate naive macrophages, which in turn help to control mycobacteria growth. Previous studies demonstrated that TNF-α and IL-12 production is differently regulated in mycobacteria infected macrophages [50]. Thus, our results provide an initial step to further understand the possible role of cytokines production in BCG cell growth inhibition as mediated by these radical scavengers.

To our knowledge, this is the first report describing the use of a radical scavenger and more specifically the first to observe the M-HA effect on mycobacterial inhibition. Among infectious diseases, tuberculosis (TB) is the leading killer with over two million casualties annually worldwide. The WHO considers tuberculosis to the most dangerous chronic disease in the world. In recent years, the emergence and spread of resistant *M*. *tuberculosis* strains has fuelled the TB epidemic by making it more difficult to treat. These results make the M-HA a potentially valuable agent, and further analyses must be performed to test it in a combination therapy with existing, well-known antimycobacterial drugs.

Regarding *P*. *aeruginosa*, the primary point of interest is an evaluation of the capacity to reduce biofilm formation. Considering that biofilm formation protects bacteria during infections, such as in chronic wounds, hospital-related pneumonia and bacterial chronic lung infections [51], the inhibition of biofilm formation by M-HA is a critically important quality as a potential therapeutic, possibly more so than its anti-microbial activity. Bacterial biofilms generally become 10–1000 times more resistant to the effects of antimicrobial agents than planktonic cells [52].

We were able to demonstrate that M-HA is capable of inhibiting *P*. *aeruginosa* biofilm formation (for an approximately 60% reduction at 5.2 μg/mL on the third day of continuous treatment); this study compared favorably with other studies that employed different anti-biofilm strategies [53]. These results were corroborated by growing *P*. *aeruginosa* cells to form a continuous biofilm in flow cells and imaging by laser scanning confocal microscopy (Table 2 and Fig. 7). Moreover, the combination of M-HA with the well-known antibiotic ciprofloxacin increased the ability to reduce an existing biofilm 10–20 times better than ciprofloxacin or M-HA alone. Using new and existing antimicrobials may provide a new strategy for bacterial therapy. Drug combinations would decrease the likelihood of resistance [54]. This finding demonstrates the feasibility of combined chemotherapy with known antibiotics for combating multi-resistant bacteria infections.

The global emergence of antibiotic-resistant strains continues unabated, along with an overall increase in the number of infections worldwide, highlighting the urgent need for new agents to treat *Mycobacterium* and *Pseudomonas* infections. Non-conventional anti-infective approaches must be explored. Our findings may provide a new basis for the discovery of new and potent radical scavenger inhibitors and improved clinical applications of these compounds in antimicrobial therapy.

## Supporting Information

S1 Fig A sequence alignment of NrdFs from *M*. *tuberculosis* (Mtub NrdF2), *M*. *bovis* subs *bovis* (Mbbo), *M*. *bovis* BCG strain Pasteur (MboP), *Salmonella typhimurium* (Styp), *E*. *coli* (Ecol) and *B*. *anthracis* (Bant). Iron ligands (ligated iron ion) and tyrosyl radical-harboring residues are shown.
(TIF)
