# Supplementary figures and images for: Methyl-Hydroxylamine as an Efficacious Antibacterial Agent That Targets the Ribonucleotide Reductase Enzyme
Source: PLoS One. 2015 Mar 17;10(3):e0122049. doi: 10.1371/journal.pone.0122049 (PMC4363900; doi:10.1371/journal.pone.0122049)

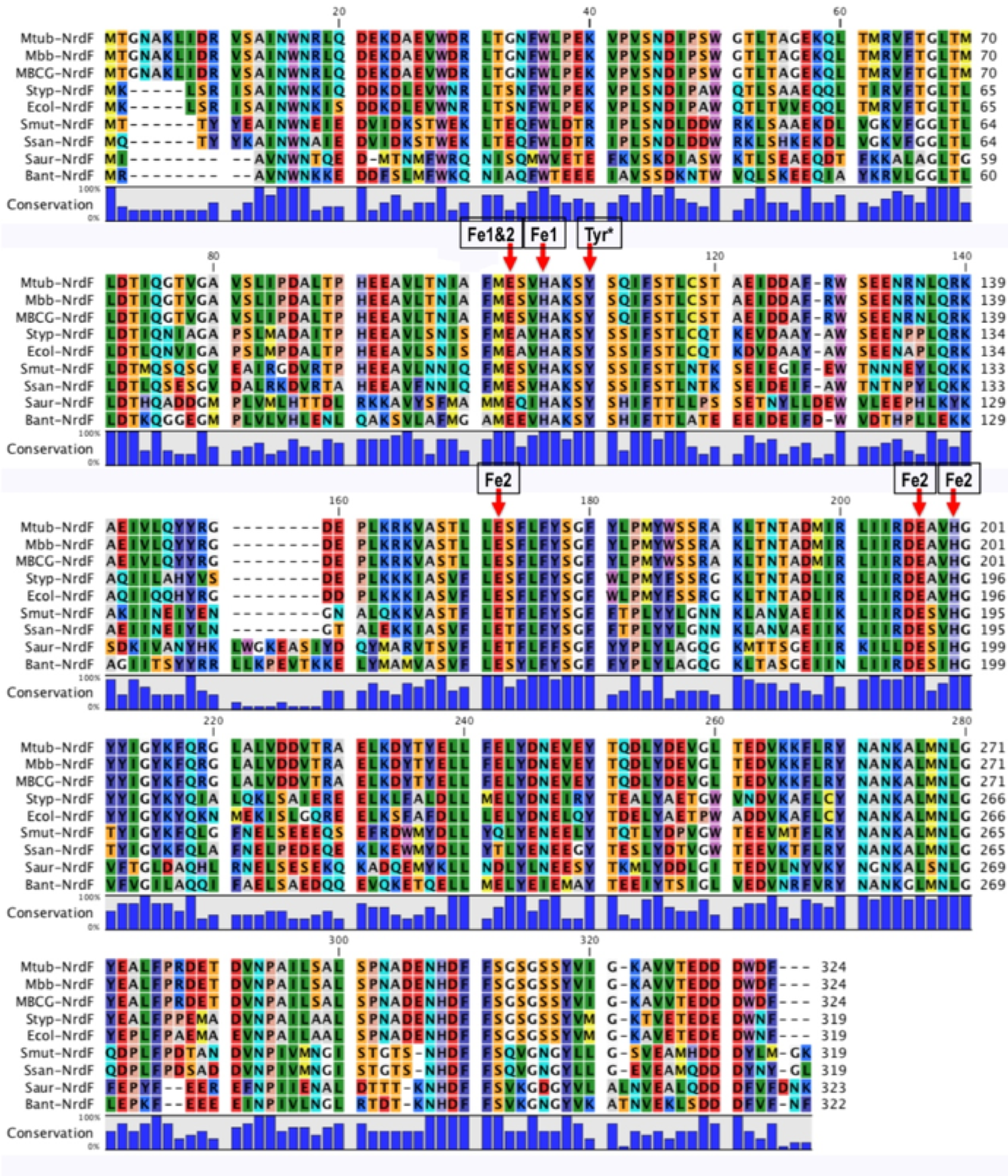

Supplement: S1 Fig — Iron ligands (ligated iron ion) and tyrosyl radical-harboring residues are shown. (TIF) [file pone.0122049.s001.tif]
